# Longitudinal Quantitative Mapping of Meniscal Morphological and Signal Intensity Differences After ACL Surgery

**DOI:** 10.1177/03635465261465318

**Published:** 2026-08-11

**Authors:** Dominique A. Barnes, Crystal J. Murray, Mohammadreza Movahhedi, Jillian E. Beveridge, Ata M. Kiapour, Martha M. Murray, Braden C. Fleming

**Affiliations:** * Department of Orthopaedics, Warren Alpert Medical School of Brown University, Providence, Rhode Island, USA; † Rhode Island Hospital, Providence, Rhode Island, USA; ‡ Department of Orthopaedic Surgery and Sports Medicine, Boston Children's Hospital, Harvard Medical School, Boston, Massachusetts, USA; Investigation performed at Boston Children's Hospital, Boston, Massachusetts, USA

**Keywords:** ACL surgery, meniscus, morphology, MRI

## Abstract

**Background:**

Meniscal tears are frequently associated with anterior cruciate ligament (ACL) injuries. Evaluating longitudinal changes in meniscal integrity using quantitative magnetic resonance imaging after ACL surgery may provide valuable insight into joint recovery and subsequent reinjury risk.

**Hypotheses:**

(1) The cross-sectional area (CSA) and normalized signal intensity (SI) profiles of the lateral and medial menisci for the surgical knees would be greater than those of the contralateral uninjured knees observed at 6, 12, and 24 months after surgery. (2) The CSA and Constructive Interference in Steady State (CISS) SI profiles obtained at 24 months would be closer to those of the contralateral limb when compared with those of the 6- and 12-month time points.

**Study Design:**

Cohort study; Level of evidence, 3.

**Methods:**

Deidentified postsurgical CISS magnetic resonance imaging scans of patients (n = 82; median age 17.6 years; 42 females and 40 males; 57 ACL restoration and 25 ACL reconstruction) were analyzed. CSA and normalized CISS SI values were extracted from the lateral and medial menisci to create morphological profiles. Statistical parametric mapping was used to compare the CSA and normalized CISS SI profiles between the surgical and contralateral limbs to identify specific regions that were different among the menisci at 6, 12, and 24 months.

**Results:**

The CSA and normalized CISS SI profiles of the anterior region of the medial menisci and lateral menisci for the surgical knees were greater than those of the contralateral uninjured knees, observed at 6, 12, and 24 months after surgery, whereas the posterior region CSA of the medial menisci was smaller. The lateral meniscus on the operated limb had a larger CSA and higher normalized CISS SI values than the lateral meniscus in the contralateral limb. The CSA and CISS SI profiles obtained at 24 months did not approach those of the contralateral limb.

**Conclusion:**

Significant changes occurred in both menisci after ACL surgery that did not return to baseline levels within 2 years. It is important to account for anatomic location when evaluating meniscal morphology in surgical knees and when evaluating the effects of ACL injuries on the surrounding tissues.

The lateral and medial menisci are vital for normal knee function and joint longevity.^17^ The menisci transmit load across the joint and decrease the stress placed on the articular cartilage.^17,28^ Studies have shown that approximately 40% to 60% of the axial load from the femur is transmitted to the meniscus.^17,20,47^ The wedge-like cross-section of the menisci allows for the tissue to modulate axial loads of the femur into hoop stresses.^11,16^ Without the menisci's ability to convert axial stress into hoop stress, greater forces are concentrated onto the articular cartilage, which may lead to its degeneration and, ultimately, the development of osteoarthritis (OA).^11,28^

Meniscal tears are frequently associated with anterior cruciate ligament (ACL) injuries.^10,18,36^ A previous study reported that concomitant meniscal injuries occurred in 61% of the patients presenting with an ACL injury, with the prevalence of concomitant lateral and medial meniscal injuries reported to be 48% and 25%, respectively.^42^ Injury to the menisci and subsequent treatment, such as partial or complete meniscectomy, have been associated with an increased risk of reinjury to the ACL and increased risk of posttraumatic osteoarthritis (PTOA) in patients who have undergone ACL reconstruction surgery.^18,31,40,46^

Recovery and risk of reinjury for patients who undergo ACL surgery have largely been tracked using patient-reported outcomes, clinical outcomes, and asymmetries in dynamic joint function.^22,39,41,52^ To supplement existing metrics, quantitative magnetic resonance imaging (qMRI) was introduced to noninvasively assess the structural properties of the ACL or ACL graft.^7,8,24,25,45,51^ Pathologic abnormalities of ligaments and grafts can be observed on qMRI as an increase in the structure's signal intensity (SI), indicating the presence of an injury or tissue degradation.^27^ Furthermore, the combination of cross-sectional area (CSA) and SI of the healing ACL has been shown to be predictive of the tissue's strength as well as revision risk within 2 years after ACL surgery.^3,5,6^ qMRI has been used to quantify meniscal healing as well.^43^ Ex vivo studies have shown that qMRI metrics can reflect morphological differences in the meniscus.^34,35^ An in vivo human study further supported that qMRI metrics, specifically UTE-T2* mapping, are sensitive to meniscal compositional changes.^9^ For this study, the SI and CSA measurements were performed on images using the Constructive Interference in Steady State (CISS) sequence. These qMRI metrics were selected for evaluation due to their clinical relevance for assessing ACL pathologies during the rehabilitation period.^3,5,6^ Evaluating longitudinal changes in meniscal integrity using qMRI after ACL surgery may provide valuable insight into joint recovery and subsequent injury risk.

The objective of this longitudinal study was to evaluate early morphologic changes of the lateral and medial menisci from qMRI scans obtained at 6, 12, and 24 months after surgery in patients who underwent either ACL reconstruction or ACL restoration (bridge-enhanced ACL restoration) surgery.^32,33^ The CSA and CISS SI profiles of the lateral and medial menisci were compared between the surgical and intact contralateral knees to identify postsurgical morphological changes in the menisci. It was hypothesized that the CSA and CISS SI profiles of the lateral and medial menisci of the surgical knees would be greater than those of the contralateral knees at all time points. It was also hypothesized that at the latest time point (24 months), the CSA and CISS SI profiles obtained would align closer to the contralateral limb than the earlier time points.

## Methods

### Participants

**Figure 1.**
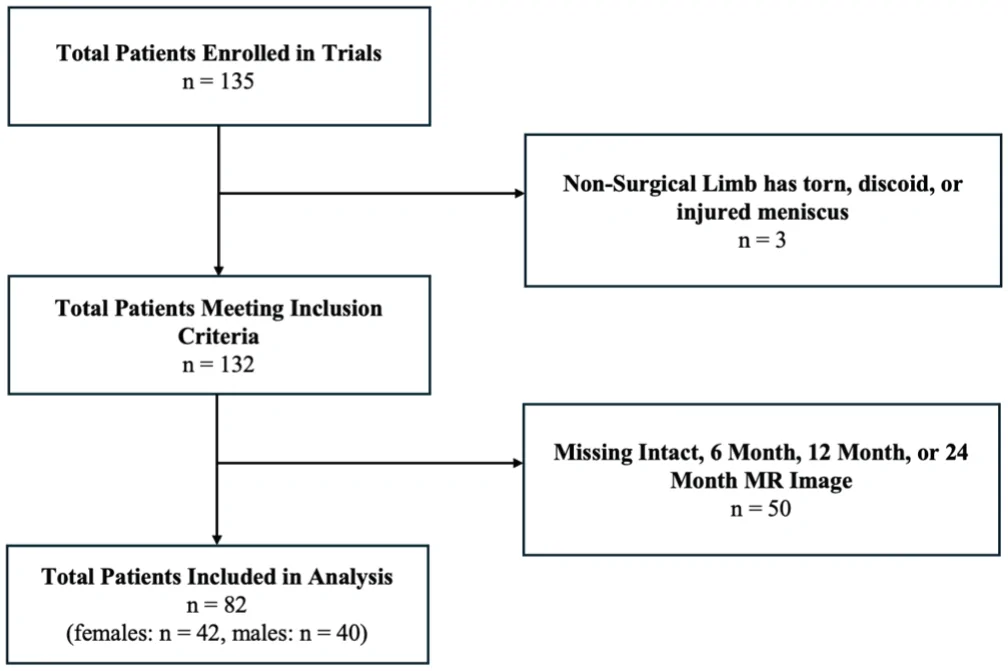
STROBE (Strengthening the Reporting of Observational Studies in Epidemiology) diagram detailing the flow of patients through the analysis. ACL, anterior cruciate ligament; MR, magnetic resonance.

Deidentified MRI scans images of patients enrolled in IRB- and FDA-approved ACL restoration trials (clinicaltrials.gov; NCT02292004; NCT02664545) were used in this study (n = 82; median age 17.6 years (range, 13-35 years); 42 females and 40 males; 57 ACL restoration and 25 ACL reconstruction) (Figure 1),^32,33^ both of which were performed at Boston Children's Hospital (IRB Nos. P00012985 and P00021470). All patients granted their informed consent. The included patients underwent unilateral ACL surgery, either ACL reconstruction or ACL restoration, and were pooled together for this analysis to increase sample size. The patients returned at 6, 12, and 24 months to undergo MRI of both knees. Patients who had a reported injured or discoid menisci in the contralateral knee at the time of MRI acquisition or were missing MRI scans at any of the 3 time points were excluded from this analysis (Figure 1).

Patients who had meniscal tears in their surgical limb were included in this analysis. Among the 82 patients included (Figure 1), 44 patients (54%) were documented arthroscopically to have a meniscal tear in their surgical limb at the time of surgery, with a total of 62 tears reported (Table 1). At the time of surgery, 36 patients presented with lateral meniscal tears in the posterior region, accounting for 44% of the patient population, with 18 counts (22% of the patient population) of posterior medial meniscal tears recorded at the same time point (Table 1).

**Table 1:** Meniscal Tear Location Details Reported at Time of Surgery*^*a*^*

Region	Lateral Meniscus	Medial Meniscus
Anterior	3	0
Middle	3	2
Posterior	36	18

a Data expressed as number of tears.

### Surgical Methods

The details of the ACL restoration and ACL reconstruction procedures have been previously reported.^32^ As mentioned above, the imaging data from the cohort used in this analysis came from 2 previously published clinical trials.^32,33^ The first trial was a nonrandomized controlled cohort study, whereas the second was a randomized controlled trial.^32,33^ Of the total number of patients included in the present analysis, 57 patients underwent ACL restoration and 25 underwent ACL reconstruction (Figure 1). For the ACL restoration procedure, a scaffold, which was composed of extracellular matrix proteins, was implanted to provide a bridge over the torn ends of the ACL.^32^ The scaffold was then saturated with the patient's blood to stimulate the ACL to heal. The ACL reconstruction was performed after a standard procedure using a quadrupled semitendinosus-gracilis autograft.^32^ A continuous-loop cortical button was used for proximal fixation and a bioabsorbable interference screw for tibial fixation.^32^

### Imaging Data

MRI scans of the knees were acquired via the 3-dimensional CISS sequence (repetition time/echo time, 14/7 ms; flip angle, 35°; field of view, 16 cm; slice × frequency × phase, 80 × 512 × 512) using a 3.0-T scanner (Tim Trio, Siemens) and a 15-channel knee coil. The images of the surgical knee were acquired at 6, 12, and 24 months. MRI scans of the contralateral knees were obtained at only 1 time point (6, 12, or 24 months) to minimize scanner time. The menisci were automatically segmented from each patient's 3D image stack using a 3D UNet-based convolutional neural network (Dice coefficient score 0.98).^48^ The automatic segmentations were verified by 2 experienced examiners using commercial image processing software (Mimics v23.0; Materialize).

### Imaging Outcomes

**Figure 2.**
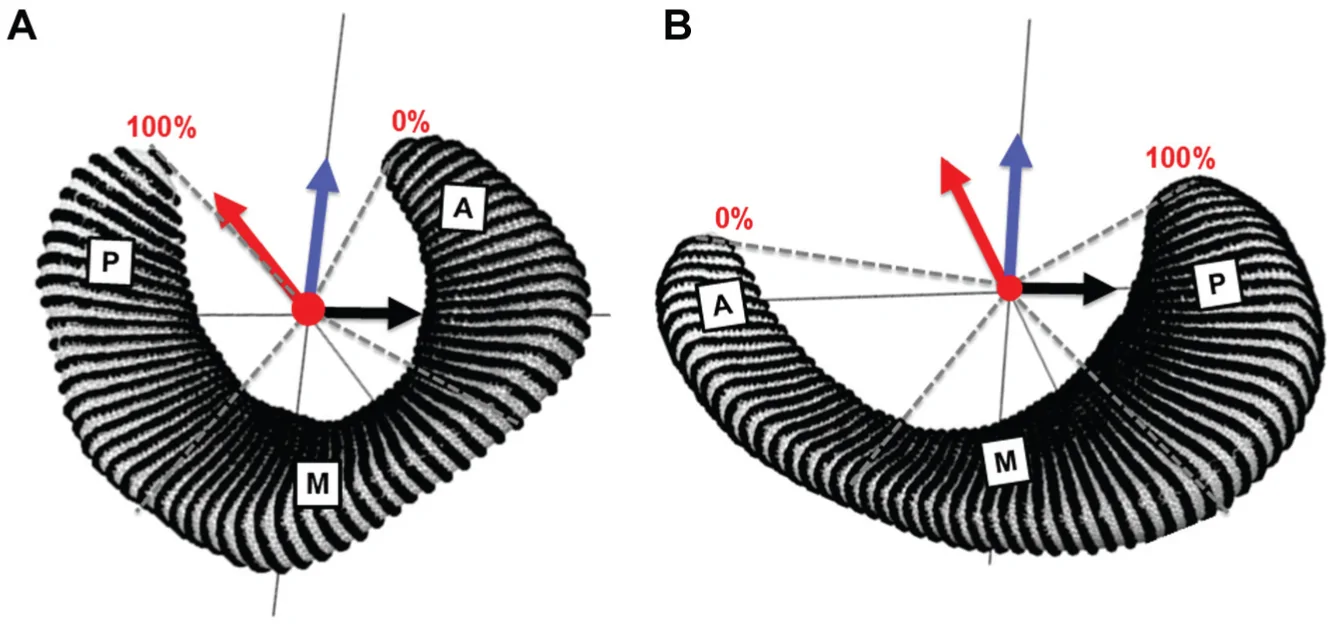
A total of 60 equally spaced cross-sectional area measurements were taken over the arc length of the (A) lateral and (B) medial menisci, normalized from 0% to 100% of the arc length from the anterior to posterior root. The gray dotted lines separate the anterior *(A)*, middle *(M)*, and posterior *(P)* regions. The meniscal coordinate system: superior-inferior axis (red), medial-lateral axis (blue), and anterior-posterior axis (black). (Adapted with permission from Barnes DA, Murray CJ, Movahhedi M, et al. Quantitative mapping of meniscus morphology using advanced imaging analysis in young patients. *Sci Rep.* 2026;16(1):15534. © The Author(s) 2026.)

The postprocessing method used to obtain the CSA and CISS SI measurements from the menisci segmentations has been described in detail.^4^ In summary, the segmented menisci were represented as 3-dimensional meshes, and their principal axes were defined using principal component analysis of the vertices.^29,30^ The coordinate systems of each meniscus were oriented such that the Z-axis corresponded to the superior-inferior direction, the Y-axis to the anterior-posterior direction, and the X-axis to the medial-lateral direction. A Taubin circle fit was applied in the midzone of each meniscus to identify the center point and standardize meniscal orientation for interpatient comparisons.^49^ For both menisci, CSA was computed at 60 equally spaced steps (~2.5° increments) along the meniscal rim arc length, normalized from anterior to posterior root, where the anterior region was considered the first third of the normalized length, the middle was the center third, and the posterior region was the final third of the normalized length (Figure 2).^4^

**Figure 3.**
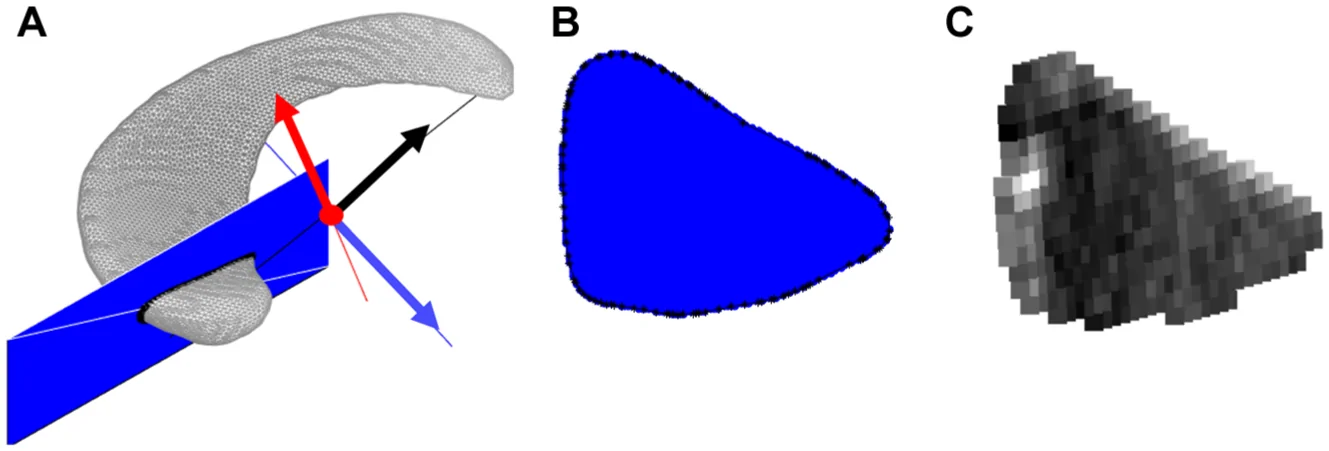
(A) Intersection of a plane to the 3D meniscus, (B) the meniscal cross-sectional area, and (C) the corresponding Constructive Interference in Steady State signal intensity values. The meniscal coordinate system: superior-inferior axis (red), medial-lateral axis (blue), and anterior-posterior axis (black). (Adapted with permission from Barnes DA, Murray CJ, Movahhedi M, et al. Quantitative mapping of meniscus morphology using advanced imaging analysis in young patients. *Sci Rep.* 2026;16(1):15534. © The Author(s) 2026.)

At each step, the CSA was calculated by intersecting a plane perpendicular to the superior-inferior axis (Z-axis), and CISS SI was obtained by calculating the mean of voxel values that fall within the intersected plane (Figure 3).^4^ Differences in CSA were quantified by subtracting the surgical limb value from the contralateral limb value for each meniscus at each step. CISS SI values were normalized for interscan variability using a previously described postprocessing protocol incorporating the femoral cortex and scaled using the background noise (Equation 1).^15^

(1) $$SI_{\mathrm{scaled}}=\mathrm{Lo}g_{2}\left(\frac{SI_{\mathrm{Meniscus}}+\;\Delta \mathrm{Noise}}{SI_{\mathrm{Bone}}+\;\Delta \mathrm{Noise}}\right).$$

### Statistical Analysis

CSA and normalized CISS SI values were extracted from both the lateral and medial menisci to generate continuous morphological profiles of each meniscus at equally spaced intervals along the entire arc length. Mean values and corresponding 95% CI values were plotted to illustrate the CSA and normalized CISS SI changes along the arc length. Statistical parametric mapping (SPM) was used to compare the CSA and normalized CISS SI profiles between the surgical and contralateral limbs. All of the patients included in this study underwent imaging of their surgical limb at 6, 12, and 24 months with the addition of the contralateral limb. The resulting SPM outputs for each meniscus highlighted specific regions that were significantly (*P* < .05) different along the arc length of each meniscus.

## Results

### Cross-Sectional Area

**Figure 4.**
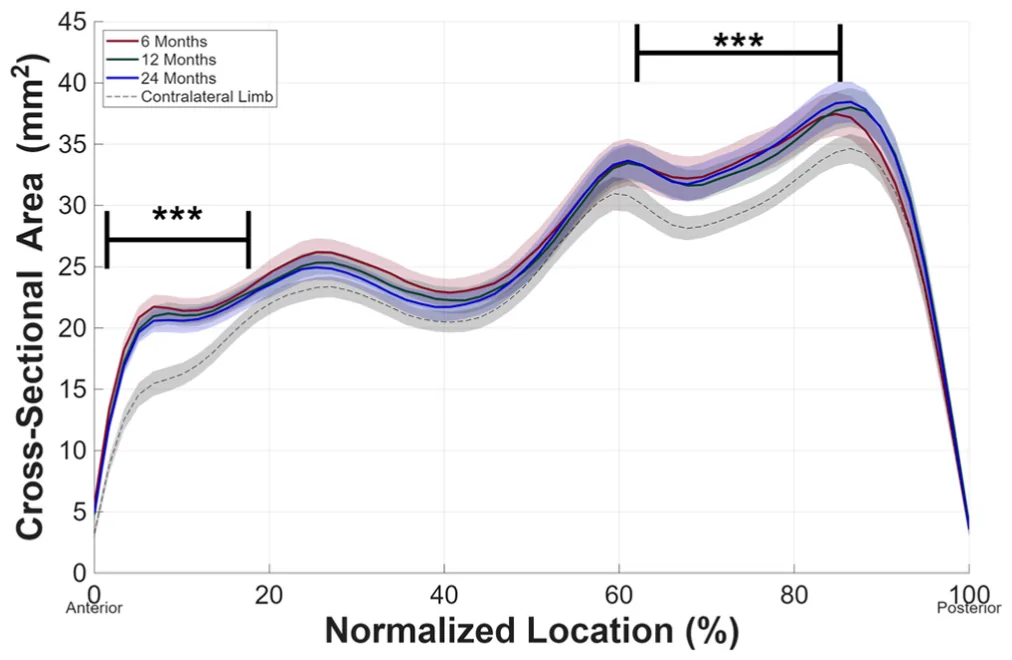
Mean and 95% CI of the lateral meniscal cross-sectional area between the contralateral limb (gray dashed) and the surgical limb at 6 months (red), 12 months (green), and 24 months (blue). Significant differences between surgical and contralateral limbs are marked with horizontal bars (***P < .001).

No significant CSA differences were observed between time points (6, 12, and 24 months) for the lateral meniscus of the surgical limb. Significant differences along the arc length of the lateral meniscus were found between the contralateral and surgical limbs (*P* < .001), specifically in the anterior and posterior regions at all time points (Figure 4). On average, the CSA was 27% larger in the anterior region of the surgical limb and 16% larger in the posterior region across all time points.

**Figure 5.**
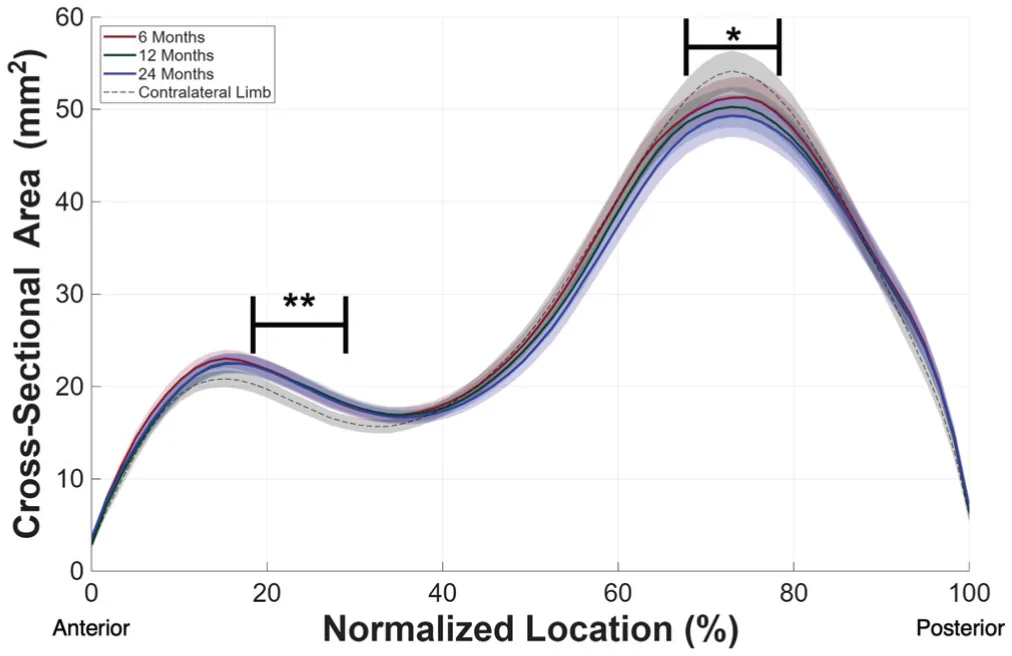
Mean and 95% CI of the medial meniscal cross-sectional area between the contralateral limb (gray dashed) and the surgical limb at 6 months (red), 12 months (green), and 24 months (blue). Significant differences between the surgical and the contralateral limbs are marked with horizontal bars (**P = .020; *P = .049).

No significant differences were observed between the time points (6, 12, and 24 months) for the medial meniscal CSA of the surgical limb. The surgical limb had a significantly larger CSA in the anterior region compared with the contralateral limb at all time points (*P* = .014) (Figure 5). On average across all time points, the CSA was 11% larger in the anterior region across all time points; however, the CSA of the posterior region was 10% smaller in the surgical limb compared with the contralateral limb at 24 months (*P* = .049).

### Normalized Signal Intensity

**Figure 6.**
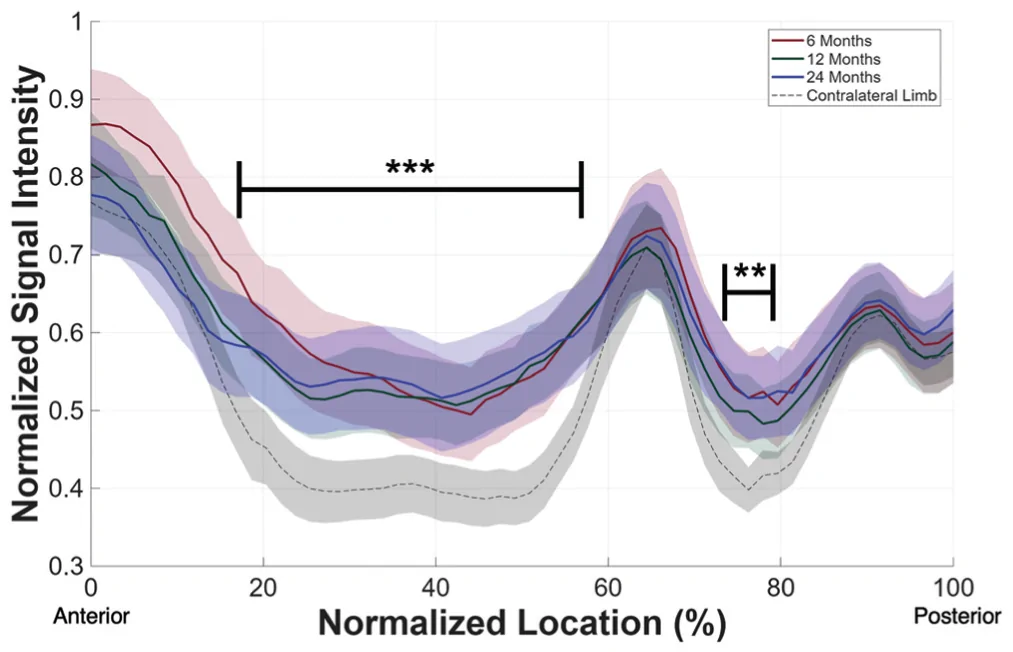
Mean and 95% CI for the normalized Constructive Interference in Steady State signal intensity of the lateral meniscus between the contralateral limb (gray dashed) and the surgical limb at 6 months (red), 12 months (green), and 24 months (blue). Significant differences between surgical and contralateral limbs are marked with horizontal bars (***P < .001; **P = .010).

No significant differences were seen in the normalized CISS SI profiles for the lateral meniscus imaged at 6, 12, and 24 months in the surgical limb. However, the normalized CISS SI values were significantly higher (*P* < .001) in the anterior, middle, and posterior regions in the surgical limb compared with the contralateral limb (Figure 6). On average, the surgical limb had a 15% higher normalized CISS SI compared with the contralateral limb in the anterior, middle, and posterior regions.

**Figure 7.**
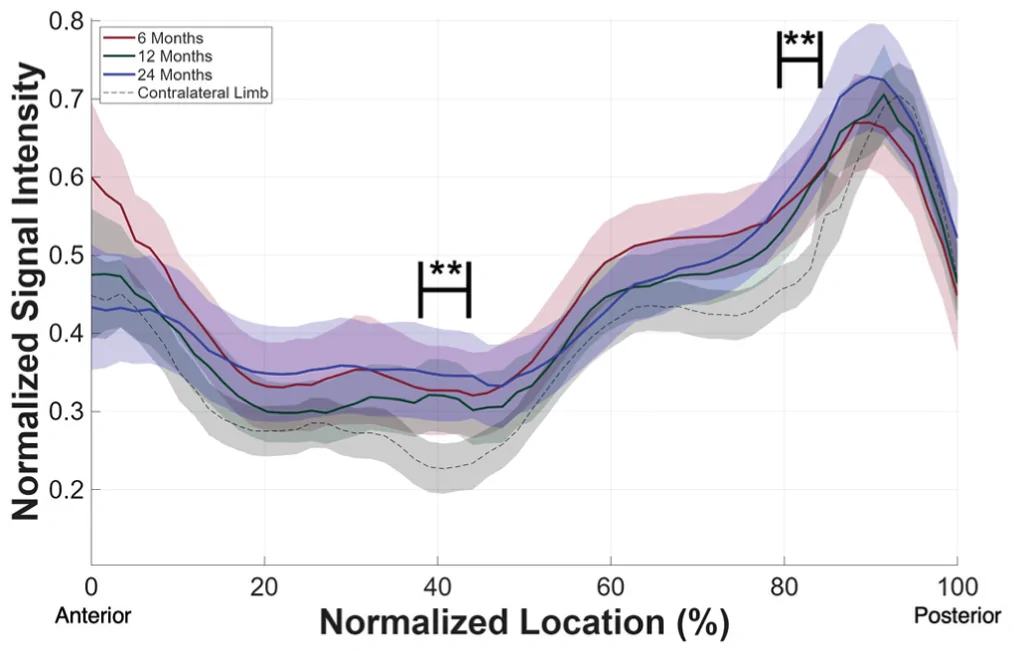
Mean and 95% CI for the normalized Constructive Interference in Steady State signal intensity of the medial meniscus between the contralateral limb (gray dashed) and the surgical limb at 6 months (red), 12 months (green), and 24 months (blue). Significant differences between surgical and contralateral limbs are marked with the horizontal bars (**P = .020).

No significant differences were detected between time points (6, 12, and 24 months) of the normalized CISS SI profiles of the surgical limbs. A significant difference was found for the normalized CISS SI in the middle and posterior regions of the medial meniscus between the surgical and contralateral limbs (Figure 7). On average, the surgical limb had a 12% higher normalized CISS SI compared with the contralateral limb in the middle and posterior regions.

## Discussion

The most important finding of the study is that there were significant differences in CSA and normalized CISS SI for both menisci of the surgical limb at all time points when compared with the contralateral limb. As hypothesized, the lateral meniscus had a larger CSA and higher normalized CISS SI values in the surgical limb, which suggests that swelling or remodeling of the tissue may occur after surgery, specifically in the anterior and posterior regions. The medial meniscus of the surgical limb had a larger CSA in the anterior region throughout time, but the posterior region was found to be smaller and had higher normalized CISS SI than the contralateral limb at 24 months. Contrary to our hypothesis, the CSA and normalized CISS SI did not return to the contralateral limb's values at 24 months and remained at levels similar to those observed at 6 and 12 months.

qMRI has been used to detect pathological abnormalities through the use of SI, as an increased SI value indicates the presence of injury or tissue degradation.^27^ Previous studies have shown the SI magnitude to be a direct representation of the severity of meniscal degeneration, where the mean SI of the medial meniscus was significantly higher in osteoarthritic knees compared with nonosteoarthritic knees.^9,19,53,55^ Comparison between meniscal changes and qMRI values has been found, such as lower T2 values being associated with complete meniscal healing,^43,54^ whereas an increase in relaxation times in multiple qMRI metric mapping techniques was found to be a measure of degeneration.^34,35^ The normalized CISS SI values reported in the current study indicate that the surgical limb menisci underwent structural property changes after the ACL injury despite surgery.^24,25,51^ These results also show that between the 6- and 24-month time points, the elevated normalized CISS SI profile and the subsequent changes to the tissue's material property did not return to uninjured levels.

The lateral meniscal CSA was larger in the surgical limb compared with the contralateral limb in the anterior and posterior regions. A preoperative MRI study of meniscal tears reported a correlation between ACL tears and lateral meniscal posterior tears.^26,38^ In that study, 95% of the patients presenting with a lateral meniscal posterior tear had an ACL injury compared with 14% of patients presenting with a medial meniscal posterior tear.^26,38^ These values align with the cohort of patients included in the current analysis, where 46% of patients presented with lateral meniscal posterior tear at the time of surgery and 22% of patients presented with medial meniscal posterior tear. The lateral meniscus plays a crucial role in restraining rotational translations.^12^ Disruption to the normal geometry of the lateral meniscus can lead to increased contact forces between the articulating cartilage surfaces during daily activity.^11^ The observed increase in CSA in the anterior and posterior regions of the lateral meniscus may reflect alterations in meniscal morphology, such as swelling or remodeling. Previous studies support the link between meniscal pathology changes after ACL injury, as injury to the posterior horn of the lateral meniscus has been associated with damage to the lateral tibial plateau and cartilage defects in the lateral femoral condyle.^21^ Our findings demonstrate persistent elevation in meniscal SI, which may reflect early alterations in the meniscal tissue that could potentially contribute to similar degenerative changes.

A primary function of the medial meniscus is to serve as a secondary stabilizer of anterior tibial translation of the knee.^18^ The demand of the medial meniscus in resisting anterior tibial loads is increased in the presence of an ACL injury or in the absence of the ACL compared with an intact knee.^1^ The anterior region of the medial meniscus is crucial for stabilizing external knee rotation, whereas the posterior region provides stabilization by absorbing approximately 50% of shear force during full extension.^16,23^ Small changes in the geometry of the medial meniscus can disproportionately elevate stress onto the articular cartilage.^13^ Previous qMRI studies have shown that qMRI relaxometry values remain high in the articular cartilage of the central medial femoral condyle compared with the contralateral side 3 years after ACL surgery.^2^ Early degenerative changes in the medial articular cartilage have also been observed at 2 years after ACLR.^50^ Small changes in the CSA of the medial meniscus may concentrate stress onto regions of the articulating surfaces, in turn causing further degeneration of the meniscal tissue that would compound any damage sustained at the time of ACL injury. This initial damage, followed by persistent changes in the structural properties and morphology of the menisci, could potentially accelerate the risk of PTOA.^53,55^ However, a direct cartilage evaluation would need to be included in future studies to make definite conclusions relating changes in meniscal CSA and CISS SI to future PTOA.

As anticipated, a substantial proportion of patients presented with concurrent meniscal injuries either at the time of ACL surgery or postoperatively. We found that 71% of patients demonstrated meniscal injury, with the majority occurring in the lateral meniscus, particularly within the posterior region. Lateral meniscal injuries remained consistently high across all time points. We noted a progressive increase in indications of medial meniscal pathology, increasing from 29% before 6 months to 31% at 12 months and 37% at 24 months. These findings are consistent with prior reports of meniscal injuries in conjunction with ACL injury.^14,37,38,44^

This study has several limitations. One inclusion criterion for this analysis was that the patient must have had their surgical limb imaged at all time points and a contralateral limb imaged at 1 time point. It should be noted that 62% of the patients included in this analysis had their contralateral limb imaged at 6 months, with the remainder at 12 and 24 months. An assumption was made that the contralateral limb would not change between 6 and 24 months. Therefore, the surgical limb at each time point was compared with the patient's contralateral limb imaged at the earliest time point available. Another limitation is that nearly half of patients with meniscal injury at the time of surgery underwent intervention at the time of surgery, most commonly meniscal repair, although partial meniscectomies and debridement were also performed. Five meniscal procedures were performed at 6 months and 3 were performed at 12 months, primarily repair. At 24 months, 6 meniscal procedures were performed, with partial meniscectomy more frequently used than meniscal repair. Thus, the variability in timing of treatment for the postoperative meniscal injuries is a study limitation, as this variability may confound longitudinal interpretation of changes in meniscal CSA and CISS SI. The age of patients included in this analysis ranged from 13 to 35 years. Therefore, the findings reported may not be generalizable to an older or aging population. Future work should incorporate a wider age range to ensure that the findings apply to a broader population. The qMRI measurement technique has inherent limitations. The MRI scans were acquired using the CISS sequence, which is not a commonly used sequence in clinical practice. The CISS sequence was selected because it provides distinct boundaries between the ACL and menisci in high-resolution images. The addition of the CISS sequence to the routine MRI knee examination would increase scan time. Work is currently underway to adapt the findings from the CISS sequence to other MRI sequences. Finally, the analysis included a relatively small sample size (n = 82). Patients from both surgical groups were pooled, which may have limited the ability to detect surgery-specific effects. Nonetheless, minor, nonsignificant differences were observed, with the ACL restoration group exhibiting lower mean normalized CISS SI and CSA compared with the ACL reconstruction group. Future large-scale studies with both surgical types would be needed to further confirm the current findings and assess the utility of CSA and CISS SI measurements of menisci in surgical knees in predicting OA.

In conclusion, this study offers new insight into the meniscal CSA and normalized CISS SI profiles in the knees of patients who have undergone ACL surgery. Significant differences were found in the CSA and normalized CISS SI of both the lateral and medial menisci, which persisted from 6 to 24 months. The lateral meniscus was found to have the greatest differences in both the CSA and normalized CISS SI between the surgical and contralateral limbs, specifically in the anterior and posterior regions. This study emphasizes the importance of accounting for anatomic location when evaluating meniscal morphology in surgical knees and the effects of ACL injuries on the surrounding tissues.
